# A rare case of acute abdomen in the adult: The intestinal duplication cyst. case report and review of the literature

**DOI:** 10.1016/j.amsu.2019.03.002

**Published:** 2019-03-12

**Authors:** Andrea Aurelio Ricciardolo, Tommaso Iaquinta, Alessandro Tarantini, Nicola Sforza, Donatella Mosca, Francesco Serra, Francesca Cabry, Roberta Gelmini

**Affiliations:** Department of Surgery, University of Modena and Reggio Emilia, Policlinico of Modena, Via del Pozzo, 71 41100, Modena, Italy

**Keywords:** Intestinal duplication, Congenital anomalies, Colonic surgery

## Abstract

**Introduction:**

Duplications of the gastrointestinal tract are rare congenital anomalies that can occur anywhere throughout the gastrointestinal tract. The reported incidence is 1/4500, and more than 80% occurs before the age of two as an acute abdomen or bowel obstruction. The most common site is Ileum (60%), while the colonic localisation is reported between 4 and 18%.

**Presentation of the case:**

Herein we report the case of a 35-year-old man, presented at the Emergency Department with fever and localised abdominal pain in the right iliac fossa. Preoperative abdominal ultrasound and CT scan showed a cystic mass of 44 × 43 × 70 mm adjoining the posterior wall of the right colon. He underwent explorative laparoscopy, laparotomy conversion, right hemicolectomy with an intra-operative diagnosis of colonic duplication cyst, confirmed by histology.

**Discussion:**

The review of the literature showed as the intestinal duplication cysts are rare congenital anomalies. The clinical presentation is variable and depends on the site and the related complications. A surgical approach based on the resection of the involved bowel tract is the treatment associated with the best long-term outcomes.

**Conclusion:**

It is important to include intestinal duplication in the differential diagnosis of acute abdomen, to ensure the best therapeutic strategy.

## Highlights

• Duplications of the gastrointestinal tract are rare congenital anomalies.
• The resection of the involved bowel tract is the treatment associated with the best long-term outcomes.
• It is important to include intestinal duplication in the differential diagnosis of acute abdomen.

## 1 Introduction

Duplications of the gastrointestinal tract are rare congenital anomalies that can occur anywhere throughout the gastrointestinal tract, from the mouth to the anus. The incidence has been reported to be 1/4500 [2]. The duplications are located on the mesenteric border of the associated native bowel and vary in size and shape: cystic in 80% of cases and tubular in the remaining 20%, with or without other congenital anomalies. They share a common wall with an adjacent portion of the gastrointestinal tract but may or may not have a communication with the bowel lumen. More than 80% of the cases present before the age of 2 years as an acute abdomen or bowel obstruction, but many duplications remain ‘silent’ unless complications occur, and therefore may not be diagnosed until adulthood. Complications include volvulus, bleeding and, rarely, malignant degeneration. The ileum is the most common site for a duplication, accounting for over 60% of cases [3]. Colonic duplication is a rare abnormality, especially in the adult [[3], [4], [5]], comprising only 6.8% of all gastrointestinal duplications [4]. Herein we report a case of colonic duplication in a 35 years old patient.

## 2 Presentation of the case

A 35-year-old man without past medical history, presented at the Emergency Department of our hospital with fever and localised abdominal pain in right iliac fossa. There was no history of vomiting and constipation.

**Fig. 1:**
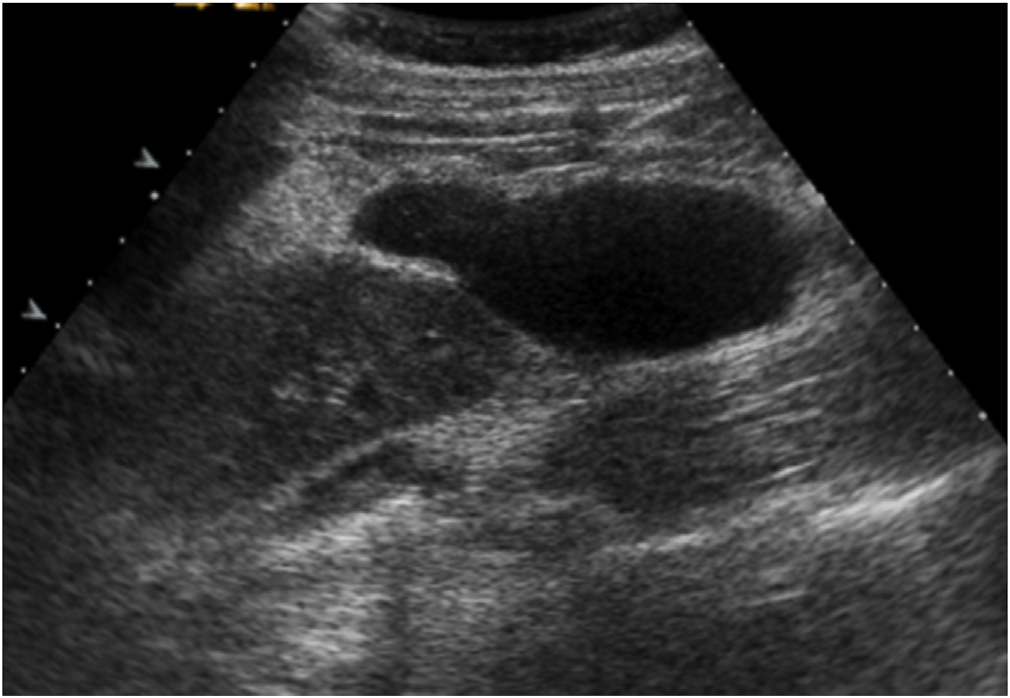
Pre-operative ultrasound.

**Fig. 2:**
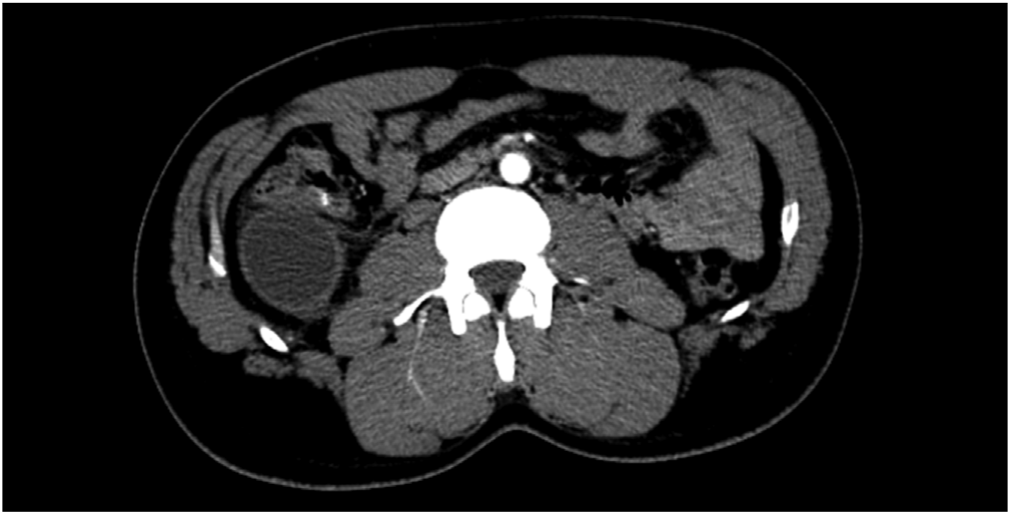
Pre-operative CT-SCAN.

**Fig. 3:**
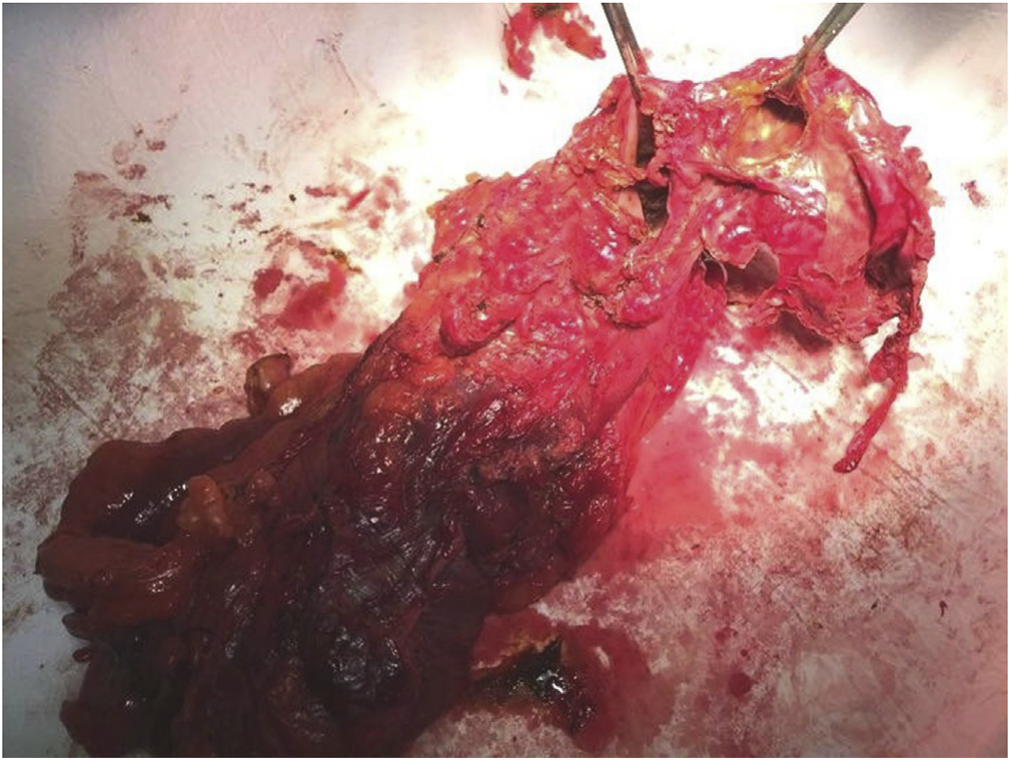
Right colon with supplication cyst.

Examination revealed moderate abdominal distention with tenderness in the right iliac fossa. The bowel movements were normal such as the complete blood count while CRP was increased (13,9 mg/dl). An abdominal ultrasound scan showed an ovoid cystic mass of 80 × 30 mm within uneven fluid content in the right side of the abdomen, close to the lower right kidney pole and the iliopsoas muscle (Fig. 1). A computed tomography (CT) scan confirmed the presence of a 44 × 43 × 70 mm structure with oval morphology, fluid content, regular but thickened walls with post-contrast enhancement, adjacent the hepatic colonic flexure (Fig. 2). Some enlarged lymph nodes were identified. The rest of the colon appeared normal. The patient was optimised for surgery, and a laparoscopic approach was decided. During the explorative laparoscopy, a cyst completely attached to the ascending colon, arising from its mesenteric border was identified. The external surface of the mass was pinkish-grey colour (Fig. 3). Laparotomy conversion was performed due to the impossibility to have a satisfying overview of the mass, and a right hemicolectomy was done. The postoperative course was regular, and the patient was discharged after six days. Pathology reported intestinal duplication cyst, communicating with the normal bowel lumen, with normal mucosa, submucosa oedema, vascular congestion and focal perivisceral inflammation. No epithelial dysplasia or malignancy was evident.

## 3 Discussion

A systematic literature review was performed using the PubMed database. The search was limited to studies on humans and those reported in the English language. The MESH search headings were used: “intestinal duplication OR duplication cyst”. The references reported in the identified studies were also used to complete the research. We have independently reviewed and screened all the papers retrieved.

Duplications of the gastrointestinal tract are rare congenital malformations that occur mostly in paediatric patients. Jejunum and ileum are the most frequent sites of localisation, while the colon is the least common site, accounting for 6.8% of all gastrointestinal duplications [4,5]. Fewer than 100 cases of colonic duplication have been reported in the literature. Complete colorectal duplication has also been described [6].

The aetiology of gastrointestinal duplications has not been well determined, while several theories have been proposed: partial twining, split notochord, environmental factors (trauma or hypoxia), embryonic diverticula and recanalisation defects [7]. Development of the myenteric plexus could be associated with cystic formation and embryogenesis of the enteric duplication [8].

They may also be associated with other anomalies of the urinary tract, genitalia and lower spinal malformations [9].

The clinical presentation is extremely variable, depending on localisation, shape, size and type of mucosa. The most are asymptomatic and discovered accidently; they may present as an acute abdomen or bowel obstruction. Complications include volvulus, bleeding and, rarely, malignant degeneration. Gastric mucosa can be found in 50.8% of cystic duplications in all locations. Peptic ulceration causing perforation or haemorrhage may occur in duplications with ectopic gastric mucosa [2].

Malignant degeneration of the duplication cyst is a rare occurrence, and most often reported in the colon. In their review of malignant tumours arising in the alimentary tract duplications, Inoue and Nakamura [10] found that, although colonic duplications account for only 6.8% of all duplications, 67% of malignancies diagnosed in these cysts occurred in colonic duplication. Adenocarcinoma is the most common histological type, followed by squamous cell carcinoma and carcinoid tumour [[11], [12], [13]].

Colonic duplication cysts can be identified using ultrasound, X-ray, CT-scan or rarely contrast enema. Colonoscopy may identify those that communicate with the wall of the colon [14]. Anyway, preoperative diagnosis is uncommon.

The paucity of reported cases does not permit a precise estimate to be made. In their experience with 78 alimentary tract duplications in 64 children over 40 years, Bower et al. [15] found ten patients with the duplication discovered at autopsy. Consequently, many duplications, without other anomalies, remain ‘silent’ until adulthood when complications occur.

The definitive diagnosis of intestinal duplication cyst has to fulfil four criteria. The duplication is a vacant structure that consists of a muscular coat, usually two layers. The cyst should be lined with epithelium that resembles that of the gastrointestinal tract. The neoformations usually extend to some portion of the alimentary tube, and they are tightly attached to it; at least the type of epithelial lining at the duplication is not necessarily consistent with that part of the gastrointestinal tract to which it is attached [13].

The treatment of symptomatic colonic duplications is en bloc resection of the cyst and adjacent viscera. Occasional small cystic duplications can be excised without colonic resection if there is no compromise of blood flow to the adjacent intestinal segment [16]. Also for the asymptomatic duplication cyst, Holcomb et al. [17] recommend, once the diagnosis is made, an elective surgical procedure should be performed to avoid complications such as perforation, bleeding, obstruction, and malignant change.

Mourra et al. reported a 7 cases series of colonic duplications in the adult, the largest one found in the literature. Four of the seven patients presented with abdominal pain, and three with symptoms of intestinal obstruction. All duplications in their series were of the cystic type; only three had a communication with the lumen. The authors claim that, although uncommon, colonic duplication should be considered in the differential diagnosis of all abdominal masses. If encountered incidentally, these lesions should be surgically addressed to avoid any future complications. Thorough sampling of the specimen is mandatory to detect malignant transformation and dysplastic lesion [18].

Fenelon et al. report a rare presentation of duplication cyst: a woman who presented with sudden onset abdominal pain underwent a CT scan that noted a calcified structure adjacent to abnormal loops of bowel. Intraoperative findings revealed an ischaemic loop of small bowel wrapped around a mass in the mesentery adjacent to the sigmoid colon. Final histology revealed a colonic duplication cyst [19].

Ohno et al. describe the case of a 15-years-old lady with a large duplication cyst occupant the lower abdomen, that communicated with the cecum, accompanied by perforated appendicitis [8].

Tufiño et al. have treated a duplication cyst attached to the cecum and the ascending colon with a laparoscopic approach. They claim laparoscopic right hemicolectomy for duplication cyst, as for malignancies, has the advantages of minimal invasion, faster recovery, and a lower rate of wound infection, and it can achieve the same degree of radicality and prognosis as open right hemicolectomy [20].

## 4 Conclusions

Colonic duplication has to be considered in the differential diagnosis of abdominal masses. Their presentation is reflective of the complications that they can cause. Pre-operative diagnosis of bowel duplication cyst is difficult; a combination of imaging studies as X-ray, ultrasounds and CT scan can be useful to show features of intestinal obstruction and the relations with contiguous organs. Surgical treatment is mandatory for all symptomatic duplication cysts and, when possible, the complete resection of the cyst and the involved bowel should be performed, to avoid future complications.

## Consent

Consent to the processing of data for scientific purposes and signed at the time of admission and kept in the medical record; the authors confirm that the patient's parents have signed consent to the publication of the data.

## Ethical approval

No ethical approval was required.

## Sources of funding

Non funding were used.

## Author contribution

Ricciardolo Andrea Aurelio, MD.

Department of Surgery, University of Modena and Reggio Emilia – Policlinico of Modena, Modena (Italy).

Via del Pozzo, 71 41100 Modena Tel: +390594223662 FAX: +390594224370.

r.andrea@hotmail.it.

Data collection, review of surgical technique literature and author of entire manuscript.

Iaquinta Tommaso, MD.

Department of Surgery, University of Modena and Reggio Emilia – Policlinico of Modena, Modena (Italy).

Via del Pozzo, 71 41100 Modena Tel: +390594223662 FAX: +390594224370.

tom91@libero.it.

Data collection, review of surgical technique literature and author of entire manuscript.

Tarantini Alessandro, MD.

Department of Surgery, University of Modena and Reggio Emilia – Policlinico of Modena, Modena (Italy).

Via del Pozzo, 71 41100 Modena Tel: +390594223662 FAX: +390594224370.

alessandro_tarantini@hotmail.it.

Data collection and co-author of case report and discussion.

Sforza Nicola, MD.

Department of Surgery, University of Modena and Reggio Emilia – Policlinico of Modena, Modena (Italy).

Via del Pozzo, 71 41100 Modena Tel: +390594223662 FAX: +39059422437.

sforza.nicola@policlinico.mo.it.

Review of surgical technique literature and co-author of discussion.

Mosca Donatella, MD.

Department of Surgery, University of Modena and Reggio Emilia – Policlinico of Modena, Modena (Italy).

Via del Pozzo, 71 41100 Modena Tel: +390594223662 FAX: +390594224370.

donatella.mosca@unimore.it.

Review of surgical technique literature and co-author of discussion.

Serra Francesco, MD.

Department of Surgery, University of Modena and Reggio Emilia – Policlinico of Modena, Modena (Italy).

Via del Pozzo, 71 41100 Modena Tel: +390594223662 FAX: +390594224370.

serrafrancescomd@gmail.com.

Data collection and author of methods and discussion.

Cabry Francesca, MD.

Department of Surgery, University of Modena and Reggio Emilia – Policlinico of Modena, Modena (Italy).

Via del Pozzo, 71 41100 Modena Tel: +390594223662 FAX: +390594224370.

francesca.cabry@unimore.it.

Review of surgical technique literature and author of introduction.

Gelmini Roberta, MD PhD.

Department of Surgery, University of Modena and Reggio Emilia – Policlinico of Modena, Modena (Italy).

Via del Pozzo, 71 41100 Modena Tel: +390594223662 FAX: +390594224370.

roberta.gelmini@unimore.it.

Supervisor and co-author of entire manuscript.

## Conflicts of interest

No conflicts of interest.

## Research registration number

The submitted case report is not a research study.

## Guarantor

Gelmini Roberta, MD PhD.

Dept. of Surgery – Policlinico of Modena, University of Modena and Reggio Emilia –

Via del Pozzo, 71 41124 Modena, Italy.

Tel: +39 0594223662.

FAX: +39 0594224370.

e_mail: roberta.gelmini@unimore.it.

## Disclosure statement

The authors have nothing to disclose.

## Provenance and peer review

Not commissioned externally peer reviewed.
